# Distinguishing magnetic and electrostatic interactions by a Kelvin probe force microscopy–magnetic force microscopy combination

**DOI:** 10.3762/bjnano.2.59

**Published:** 2011-09-07

**Authors:** Miriam Jaafar, Oscar Iglesias-Freire, Luis Serrano-Ramón, Manuel Ricardo Ibarra, Jose Maria de Teresa, Agustina Asenjo

**Affiliations:** 1 Instituto de Ciencia de Materiales de Madrid, CSIC, Madrid, 28049, Spain; 2 Dpto. Física de la Materia Condensada, Universidad Autónoma de Madrid, Madrid, 28049, Spain; 3 Instituto de Ciencia de Materiales de Aragón, Universidad de Zaragoza-CSIC, Zaragoza, 50009, Spain; 4 Dpto. Física de la Materia Condensada, Universidad de Zaragoza-CSIC, Zaragoza, 50009, Spain; 5 Laboratorio de Microscopías Avanzadas (LMA), Instituto de Nanociencia de Aragόn (INA), Universidad de Zaragoza, Zaragoza, 50018, Spain

**Keywords:** electrostatic interaction, focused electron beam induced deposition, Kelvin probe force microscopy, magnetic force microscopy, magnetic nanostructures

## Abstract

The most outstanding feature of scanning force microscopy (SFM) is its capability to detect various different short and long range interactions. In particular, magnetic force microscopy (MFM) is used to characterize the domain configuration in ferromagnetic materials such as thin films grown by physical techniques or ferromagnetic nanostructures. It is a usual procedure to separate the topography and the magnetic signal by scanning at a lift distance of 25–50 nm such that the long range tip–sample interactions dominate. Nowadays, MFM is becoming a valuable technique to detect weak magnetic fields arising from low dimensional complex systems such as organic nanomagnets, superparamagnetic nanoparticles, carbon-based materials, etc. In all these cases, the magnetic nanocomponents and the substrate supporting them present quite different electronic behavior, i.e., they exhibit large surface potential differences causing heterogeneous electrostatic interaction between the tip and the sample that could be interpreted as a magnetic interaction. To distinguish clearly the origin of the tip–sample forces we propose to use a combination of Kelvin probe force microscopy (KPFM) and MFM. The KPFM technique allows us to compensate in real time the electrostatic forces between the tip and the sample by minimizing the electrostatic contribution to the frequency shift signal. This is a great challenge in samples with low magnetic moment. In this work we studied an array of Co nanostructures that exhibit high electrostatic interaction with the MFM tip. Thanks to the use of the KPFM/MFM system we were able to separate the electric and magnetic interactions between the tip and the sample.

## Introduction

The most valuable asset of scanning force microscopy (SFM) is its versatility for studying a variety of interactions between the tip and the sample surface [1–3]. The SFM techniques can be used to detect different short, medium and long range interactions with high sensitivity and lateral resolution. The spreading of this technique was possible thanks to the development of specific operation modes and to the functionalization of the probes. Thus, regarding the mode employed, SFM can be used to characterize the topography of organic and inorganic materials and to study chemical (composition), mechanical (including friction and stiffness, etc.), electrical (surface potential, work function), magnetic (domain structure) or biological (specific recognition) properties. A priori, the unknown contribution of every kind of force to the total force measured leads to serious problems for obtaining quantitative information from the measurements [4].

Among those SFM techniques, magnetic force microscopy (MFM) [5] was developed to characterize the domain configuration of ferromagnetic thin films, rather than the surface of the bulk materials, and it has been intensively used to characterize magnetic nanostructures. However, MFM is nowadays proposed as a valuable technique to characterize more complex systems such as organic nanomagnets [6], magnetic oxide films [7], superparamagnetic particles [8–9] and carbon based materials [10–11]. In general, these materials present low magnetic moment at room temperature. In addition, since the substrate and the nanomagnets present quite different electronic behavior, the sample can exhibit large surface potential differences, which cause heterogeneous electrostatic interactions between the tip and sample along the surface [12–13]. Notice that all of the tip–sample interactions provoke changes in the total force, i.e., they modify the cantilever state. In MFM it is a usual procedure to separate the topography and the magnetic signal by scanning at a certain height such that that the long range tip–sample interactions dominate. An additional problem appears if several different long range interactions are present between the tip and sample. In such cases, two different methods to distinguish clearly the origin of the forces can be proposed: (i) By applying in situ a magnetic field during the MFM operation [14–16]; (ii) performing a combination of Kelvin probe force microscopy (KPFM) [17–18] and MFM to compensate the electrostatic contribution to the frequency shift signal. In the first method the evolution of the MFM signal with the magnetic field is a signature of the magnetic character of the sample. In addition, by means of variable field MFM [19], the changes in the signal as a function of the external magnetic field can be utilized either to evaluate the coercivity of the MFM probes [20–21] or to analyze the magnetic behavior of micro- and nanostructures [22–23], depending on the values of both the tip and sample coercive fields (*H*_tip_ and *H*_sample_) and the maximum external magnetic field applied (*H*_max_). Notice that the MFM measurements under an external magnetic field allow us to state the origin of the interaction but cannot remove other interactions from the magnetic signal in the case that they exist. However, the second method proposed, the KPFM/MFM combination, which was recently used to obtain an upper bound for the force gradient produced by a possible magnetic signal in graphite [24], allows us to nullify the main electrostatic interaction between the tip and the sample. Few works have been published on this topic despite its crucial importance in the study of new nanomagnet elements with weak magnetic signal and where, in general, the surface presents heterogeneous composition and electrical behavior.

### Tip–sample interactions

When a magnetic (and in general conductive) tip approaches the sample, different mutual interactions are possible [25]: Long range electrostatic (*F*_e_) and magnetic forces (*F*_m_), medium range van der Waals interactions (*F*_vdW_), or short range chemical interactions. Assuming that the short range interactions are negligible at the distances used for MFM, the total force between the tip and the sample (*F*_t_) is:

[1]
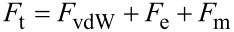


The van der Waals [26] force between a spherical tip and a semi-infinite flat sample can be written as:

[2]
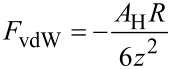


where *A*_H_ is the Hamaker constant that depends on the material, *R* is the tip radius and *z* is the tip–sample distance. When both the tip and the sample are conductive and there is an electrostatic potential difference (*U*) between them, the electrostatic force [27–28] is

[3]
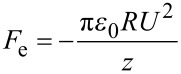


where *R* is the radius of the metallic part of the spherical tip, *ε*_0_ is the permittivity of free space and *z* is the effective tip–sample distance taking into account the oxide layer. Regarding the magnetic force, there are widely used models for the magnetic tip–sample interaction, which can be fitted to the experimental data [29], but no simple, well-established function. We can obtain an order of magnitude estimation simply by modeling both the tip and the sample as magnetic dipoles and, hence, the magnetic force is proportional to the magnetic moment of both the tip and sample (*m*_tip_ and *m*_sam_) [22] and decays with the distance as *z*^4^ [30].

Typical values of the three components of the force for three tip–sample distances are displayed in Table 1. The values have been calculated using Equation 2 and Equation 3 and the equation in [30]. For the van der Waals forces we assume a tip radius of 30 nm and *A*_H_ of about 10^−19^ J. The electrostatic interaction is calculated for a tip with an electrical radius slightly smaller due to the existence of an oxide layer 2 nm thick and a contact potential between tip and sample of 1 V [25]. We calculate the magnetic interaction of two Co spheres with a radius of 20 nm. The values in Table 1 show that at short distances all the interactions are on the same order of magnitude, although van der Waals interaction dominates at distances below 1 nm. At the typical tip–sample distance during the MFM imaging, around 30 nm, the *F*_vdW_ can be negligible but the *F*_e_ and *F*_m_ remain comparable.

**Table 1:** Values of the *F*_vdW_, *F*_e_ and *F*_m_ for three different tip–sample distances, *d*^a^.

	*F*_vdW_ [nN]	*F*_e_ [nN]	*F*_m_ [nN]

*d* = 30 nm	5.0 × 10^−4^	2.3 × 10^−2^	5.7 × 10^−2^
*d* = 2 nm	1.2 × 10^−1^	1.3 × 10^−1^	4.4 × 10^−1^
*d* = 1 nm	5.0 × 10^−1^	1.6 × 10^−1^	4.9 × 10^−1^

^a^The value of *z* corresponds to *d* in the case of *F*_vdW_; for the *F*_e_ case *z = d* + 2 nm due to the existence of an oxide layer; and for *F*_m_ *z = d* + 40 nm due to the position of the dipole centers.

To avoid a contribution of the short and medium range interactions to the total tip–sample force in MFM, the images are recorded at a given distance from the surface using the so-called lift mode [31] or retrace mode [32]. Typical distances for this second scan are between 20 nm and 50 nm. However, in order to improve both the lateral resolution and sensitivity, especially when dealing with materials with weak magnetization (either of the tip or the sample), it is crucial to keep the tip–sample distance as small as possible. Thus, a balance has to be found in order to avoid the van der Waals contribution and to simultaneously improve the magnetic signal. Another important issue, that has conveniently been neglected so far, is how to distinguish between the magnetic and the electrostatic interaction in certain kinds of samples. These long range interactions can have similar values in the range of a few tens of nanometers, as shown in Table 1. Since, in a first approximation, the magnetic force is proportional to the sample and tip magnetic moments, samples with high magnetization generate stronger stray fields and the magnetic interaction dominates over the electrostatic one. In such cases, the electrostatic force can be neglected, which is the usual procedure in standard MFM measurements [5]. However, it is well known that an electrostatic interaction is present whenever tip and sample exhibit a different work function. For homogeneous samples, the work function difference can be compensated by applying an appropriate bias voltage and, hence, an unambiguous magnetic image can be obtained [33]. Sometimes, this effect induces superposition of magnetic and topographic contrast in a MFM image [34]. In the heterogeneous sample case, it is impossible to compensate the electrostatic force with a single fixed bias voltage since it depends on the (*x*,*y*) position, and it is then necessary to use KPFM techniques. If the electrostatic interactions are not compensated, an incorrect interpretation of the MFM could be made. This is especially problematic in samples with low magnetic moment where it is crucial to distinguish clearly the origin of the interaction for a correct interpretation of the results [10].

## Results and Discussion

In the present work we have studied cobalt nanowires grown by focused-electron-beam-induced deposition (FEBID). The sample growth was performed in a commercial dual beam^®^ equipment using a field emission scanning electron microscope with Co_2_(CO)_8_ as gas precursor. The substrate material used in all the samples studied in this paper is As-doped (n-type) Si(111). Different nano- or submicrometric structures were grown for this experiment: (i) Co straight wires 5 μm long, 500 nm wide and a thickness ranging from 10 nm to 400 nm; (ii) Co L-shaped wires with long arm of 10 μm and short arm of 5 μm, the width of the wires varies between 125 nm and 2 μm, and the thickness between 50 nm and 200 nm.

An appropriate selection of the growth parameters leads to high-purity deposits (over 95% Co) with magnetic properties similar to those of bulk cobalt [35] and good domain wall conduit behavior [36]. All the structures presented in this study were deposited with an electron beam current of 2.1 nA, an acceleration voltage of 10 kV and 1 µs dwell time. The nanowires grown by this technique are polycrystalline with grain sizes of a few nanometers oriented randomly, thus shape anisotropy is the main magnetic energy contribution [37] that controls their domain wall structure and magnetization reversal process [38].

As we were using a semiconductor material as a substrate, we expected that some charging effects would appear where the electron beam was scanned. The secondary electrons generated when the electron beam impinges on the substrate may not have enough energy to overcome the work function of the surface and penetrate the bulk and as a consequence they will become trapped in the neighboring area of the wires. During the FEBID deposition process some secondary electrons reach the substrate surface near the scanning area, even at distances of more than 1 μm, with energy enough to partially decompose the precursor gas molecules, producing a parasitic deposit, or a so-called “halo”. The number of secondary electrons that reach the surface near the sample area is less than in the scanning area, and on average less energetic. Therefore, the decomposition of the precursor gas (Co_2_(CO)_8_) in the halo is not complete. As a consequence, the halo is an insulating material of which the major components are C and O (the Co content in the halo is lower than 20% in our system). Previous works have reported similar results with respect to the Co content of the halo [39]. Secondary electrons generated during the growth may get trapped in the halo, increasing the surface potential. On the other hand, a thin native oxide layer covers the Co thin film the moment the samples are exposed to the atmosphere, with a thickness of around 2 nm. These insulating side effects enhance the accumulation of charge in the area of the deposits, thus changing the electrostatic potential of the area close to where the electron beam has been scanned.

The measurements were performed with a commercial magnetic force microscope from Nanotec Electronica S. L., and the images were processed with WSxM [40]. This system has been conveniently modified to apply in situ in-plane and out-of-plane magnetic fields [14]. Since the electric field can also be varied continuously, this system can be used to obtain high resolution SPM images of individual nanostructures under continuously applied electric and/or magnetic fields. The probes used in this experiment are commercial Si cantilevers (nanosensors PPP-FMR, *k* = 1.5 N/m and *f* = 75 kHz) coated with a Co/Cr sputtered thin film. The thickness of the Co coating (25 nm) was selected to prevent the influence of the tip stray field on the magnetic state of the sample. Before each experiment the probes were magnetized along their pyramidal axis and their magnetic behavior was analyzed under an in situ magnetic field [21]. In this particular case, we have prepared probes with an in-plane coercive field higher than the magnetic field values to be applied in the experiments. In addition, micromagnetic simulations have been performed by means of the object oriented micromagnetic framework (OOMMF) code [41] and with the polycrystalline cobalt values [37] and a cell size of 5 nm.

**Figure 1:**
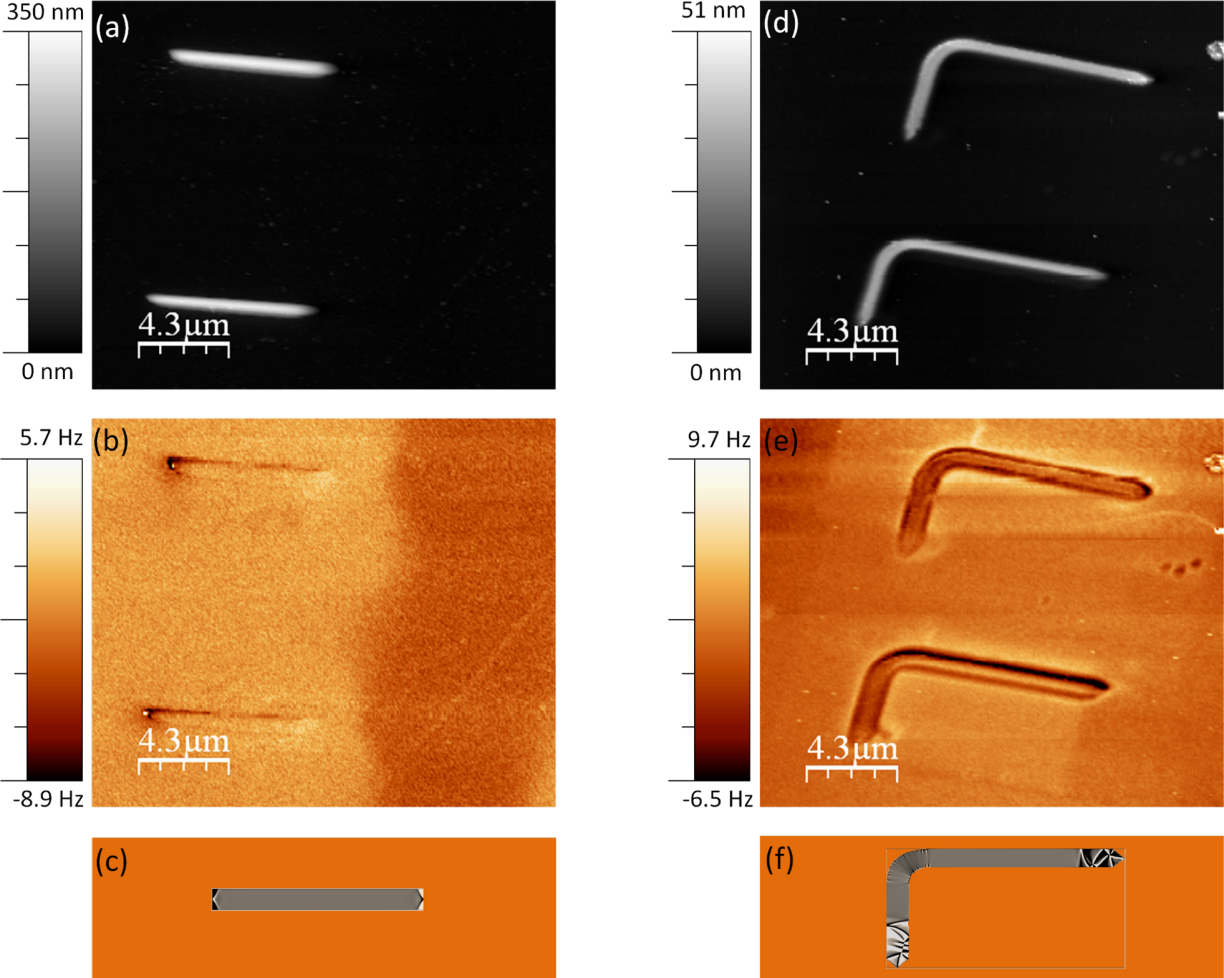
(a) Topography and (b) frequency shift images corresponding to the Co wires; (d) topography and (e) frequency shift images corresponding to the L-shape nanostructure. The frequency shift images were acquired at a retrace distance of 30 nm with *V*_bias_ = 0V. Cantilever amplitude: (a–b) *A* = 5 nm and (d–e) *A* = 8.5 nm. Simulated maps of the magnetization distribution (divM) obtained by OOMMF code of the Co wire (c) and L*-*shape Co nanostructure (f) in the remanent state after saturation along the main axis of the elements.

As usual procedure in MFM, we record two images simultaneously, the topography, obtained at small tip–sample distance, and the frequency shift, which is obtained at a retrace distance of 30 nm. Figure 1a and Figure 1b shows the topography and the frequency shift images of the Co wires. Figure 1c corresponds to the magnetization divergence (DivM) obtained by OOMMF. Such a magnetic distribution, the so-called “dipolar contrast”, is for a remanent state after saturating the wire by applying 10 kOe along the axis. This contrast, which is still observed on the images of the Co wires in Figure 1b, is typical of the single domain structures. Surprisingly, the image corresponding to the experimental magnetic signal shows an additional area of high signal surrounding the wire that should not correspond to any kind of magnetic interaction since it is measured outside of the Co nanostructure. Co L-shape nanostructures were also studied by MFM (Figure 1e) and modeled by OOMMF code (Figure 1f). Yet again, the frequency shift image displayed in Figure 1e does not correspond to the expected MFM image, which should be similar to the divM map in Figure 1f. Moreover, the magnetic signal seems to be completely masked by other long range interactions, i.e., the electrostatic forces. These kinds of images can be erroneously interpreted as magnetic contrast in the case of complex magnetic materials.

**Figure 2:**
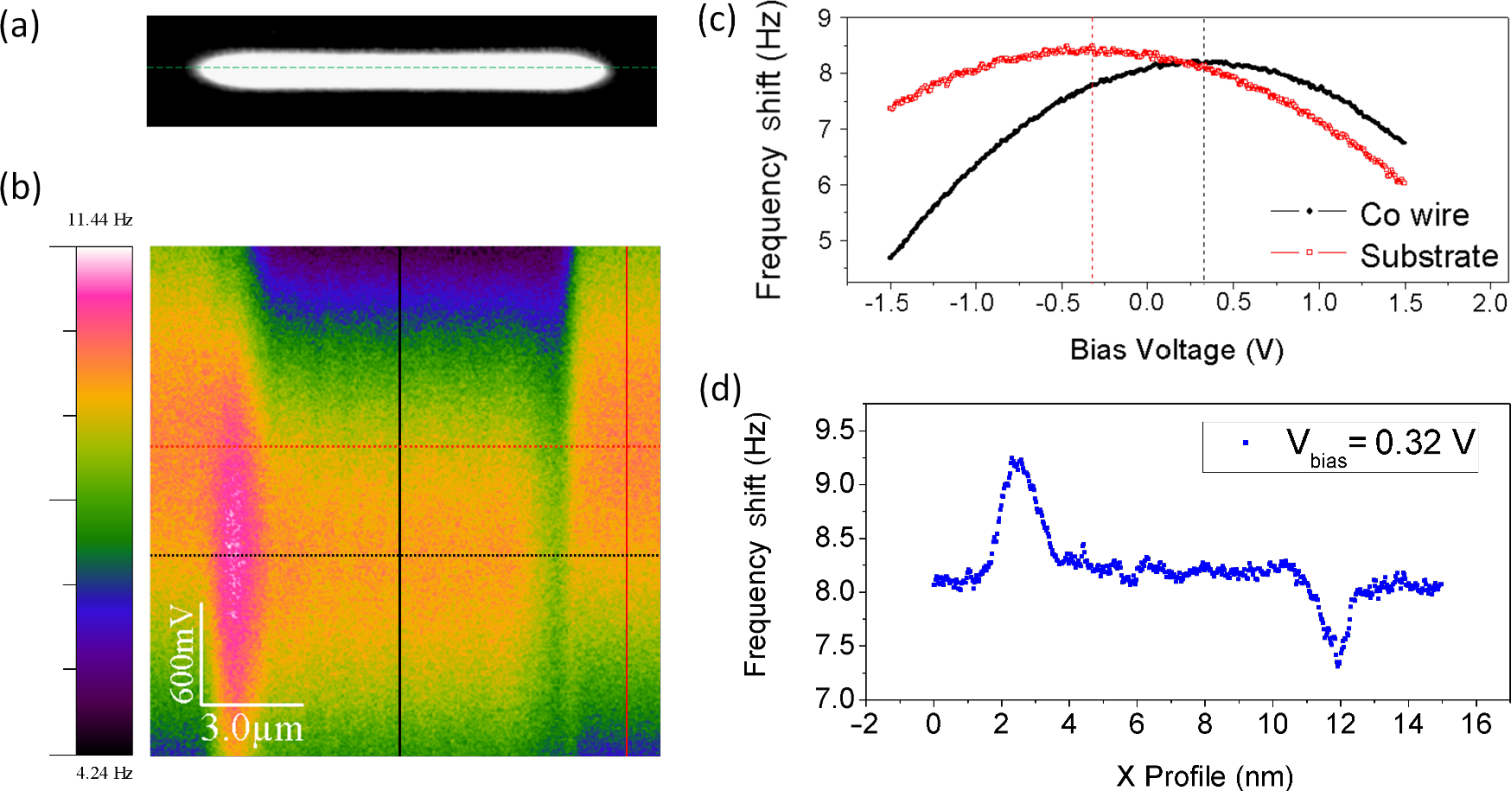
(a) Topography of the Co wire. The dashed line corresponds to continuous scanning along the profile while varying the bias voltage. (b) Frequency shift signal measured in the 3D mode (acquired at a distance of 100 nm). The fast scan corresponds to the *x*-axis scan all along the main axis of the Co wire and the slow scan is the bias voltage applied between the tip and the sample. (c) Frequency shift curves measured along the vertical profiles marked in (b) that correspond to the Co wire (black line) and substrate (red line). (d) Frequency shift measured along the wire at *V*_bias_ = 320 mV (horizontal dashed black line marked in Figure 2b). The oscillation amplitude was *A* = 7 nm and the scan rate was 1 Hz.

In order to determine the origin of this contrast we varied the electric field between the tip and the sample. Instead of recording images at different bias voltage, we use a more useful technique to characterize the electrostatic behaviour of the samples, the so-called 3D modes [42]. This mode is based on measuring a signal (or a set of signals) while two parameters vary along the fast and slow scans. In our case, we measured the frequency shift (at 30 nm above the surface) while keeping the tip scanning along a selected profile (fast scan; all along the main axis of the wire marked in Figure 2a) and varying the bias voltage (slow scan). Figure 2b shows the frequency shift signal measured along a Co wire (with an MFM probe) as the bias voltage was varied between ±1.5 V. The vertical profiles measured on the Co nanowire (black line) and on the substrate (red line) are shown in Figure 2c. Notice the parabolic dependence of the frequency shift versus voltage, which corresponds to an electrostatic interaction between the tip and the sample [43]. The bias voltage at the apexes of those parabolas, measured in different regions of the sample, corresponds to the contact potential between the tip and the selected region of the sample. The respective maxima of the curves in Figure 2c are shifted to about +320 mV when the tip is on top of the Co wire and to about −320 mV in the case of the Si substrate. Thus, according to these results, by measuring the frequency shift on top of the Co wire at *V*_bias_ = 320 mV (horizontal black dashed line) we should detect only the magnetic signal without any electrostatic interaction between the tip and this particular region of the sample. Indeed, this measurement is represented in Figure 2d. In this curve we observe the typical dipolar contrast (positive in one extreme and negative in the opposite one) corresponding to a single domain nanostructure.

**Figure 3:**
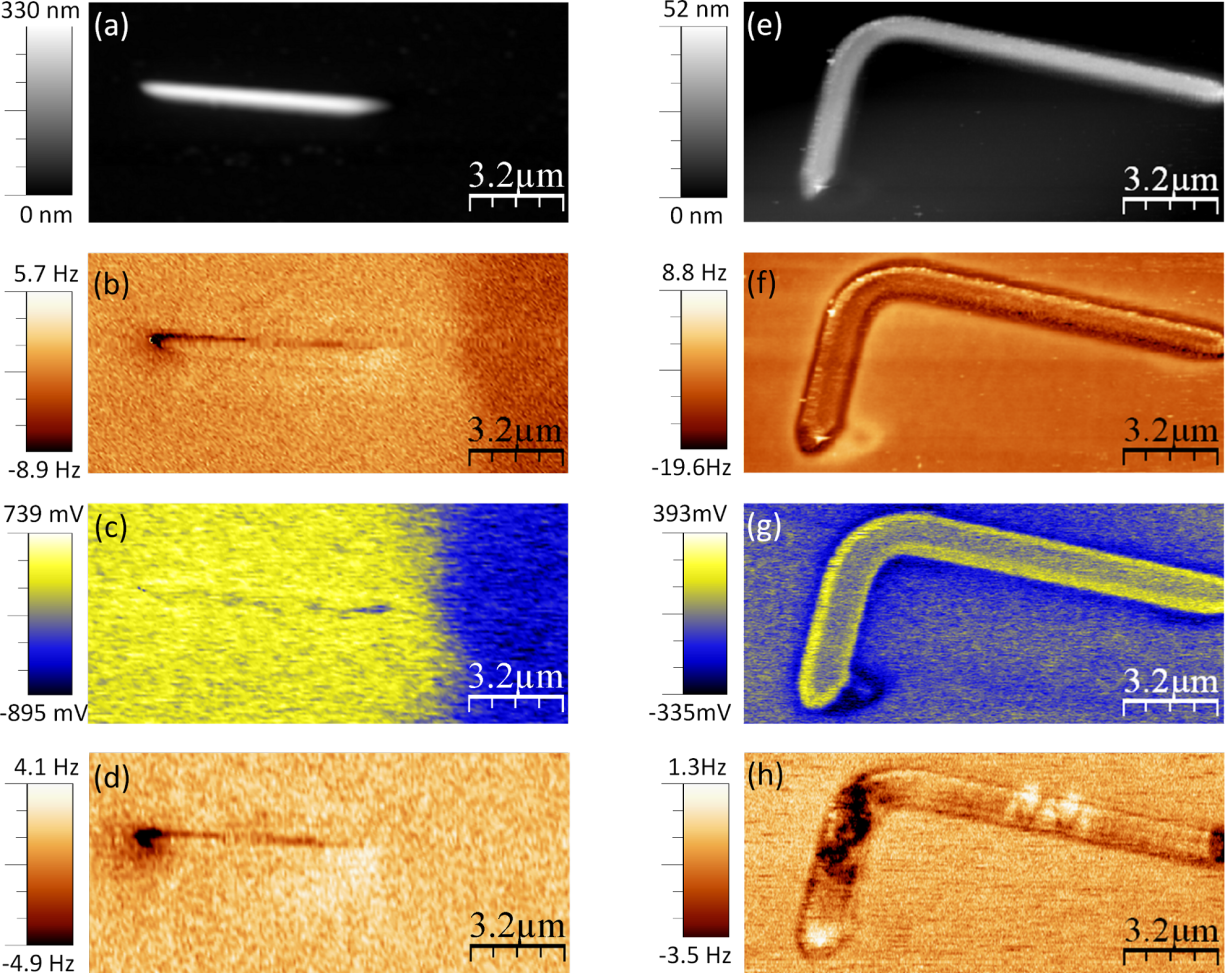
Topography of (a) Co nanowires and (e) L-shaped Co nanostructure. (b) and (f) frequency shift images measured without KPFM acquired at a retrace distance of 30 nm and 25 nm respectively. (c) and (g) surface potential images obtained by the KPFM technique. (d) and (h) MFM images (frequency shift) of the Co nanostructures measured when the KPFM bias correction was switched on. The oscillation amplitudes were (a–d) *A* = 5 nm and (e–h) *A* = 8.5 nm.

The 3D mode technique presented in this work is a highly valuable method to ascertain the electrostatic origin of some component of the frequency shift signal measured on magnetic elements. However, in this kind of system it is impossible to cancel the electrostatic force everywhere during scanning at a single, fixed bias voltage. Nevertheless, KPFM allows us to cancel the electrostatic force at every point of the image by applying the correct compensation voltage (*V*_dc_) at each (*x*,*y*) position, and hence it is the only method that can be used to unambiguously measure the magnetic signal. The KPFM/MFM results are presented in Figure 3. The images in Figure 3a and Figure 3e (similar to the data in Figure 1a and Figure 1c) correspond to the topography of the nanowires. The frequency shift images shown in Figure 3b and Figure 3f (zooms of the Figure 1b and Figure 1d respectively) were measured at 30 nm without the KPFM bias correction. Notice that the magnetic information is largely masked by the electrostatic signal. However, by using the KPFM/MFM combination, that is, activating the KPFM bias correction during the MFM operation, we were able to separate the electrostatic contribution (Figure 3c and Figure 3g) and the magnetic signal (shown in Figure 3d and Figure 3h).

It is important to note that the electrostatic interaction can also affect the topographic images [44]. In the experiments presented here for these rather thick structures this effect was not significant. Height differences less than 1 nm (a deviation about 2%) were found when we measured the topography of the same structure with and without activation of the KPFM mode (more details in Supporting Information File 1). After removing the electrostatic interaction from the MFM signal, we can apply a magnetic field to study the magnetization process of a single structure. As an example, in Supporting Information File 2 we present a combination of KPFM/MFM under in situ magnetic field on a single L-shaped nanostructure. The initial state of the sample which corresponds to the images in Figure 3 is “as-prepared”. Similar L-shaped structures were previously studied through the Magneto-Optical Kerr Effect [36] and good domain wall conduit was found (lower domain-wall propagation field than nucleation field). Using this technique, it has therefore been possible to obtain additional valuable information about the type of domain walls that form and propagate along the wires.

## Conclusion

In this work we have shown that different tip–sample interactions are present when a magnetic (and also conductive) tip approaches the magnetic sample. These interactions have comparable values regarding the electric and magnetic properties of the system at the same tip–sample distances. When a heterogeneous sample (as is the case of nanostructures deposited on a substrate) is studied, and especially in the case of low magnetic moment materials, it is necessary to be aware of this problem in order to prevent incorrect image interpretation, examples of which can indeed be found in the literature.

To avoid mistakes in the interpretation of the MFM images it is crucial to distinguish between the separate contributions to the frequency shift signal by varying the external magnetic and electric fields. These methods allow us to elucidate the origin of the signal or the presence of different components. However, only by means of KPFM and MFM in combination is it possible to cancel the electrostatic interaction between the tip and sample at every point in the image, thus obtaining a pure magnetic signal. Thus, the KPFM/MFM combination is a powerful technique that allows us to obtain unambiguous magnetic images of low magnetic moment materials.

## Experimental

**Figure 4:**
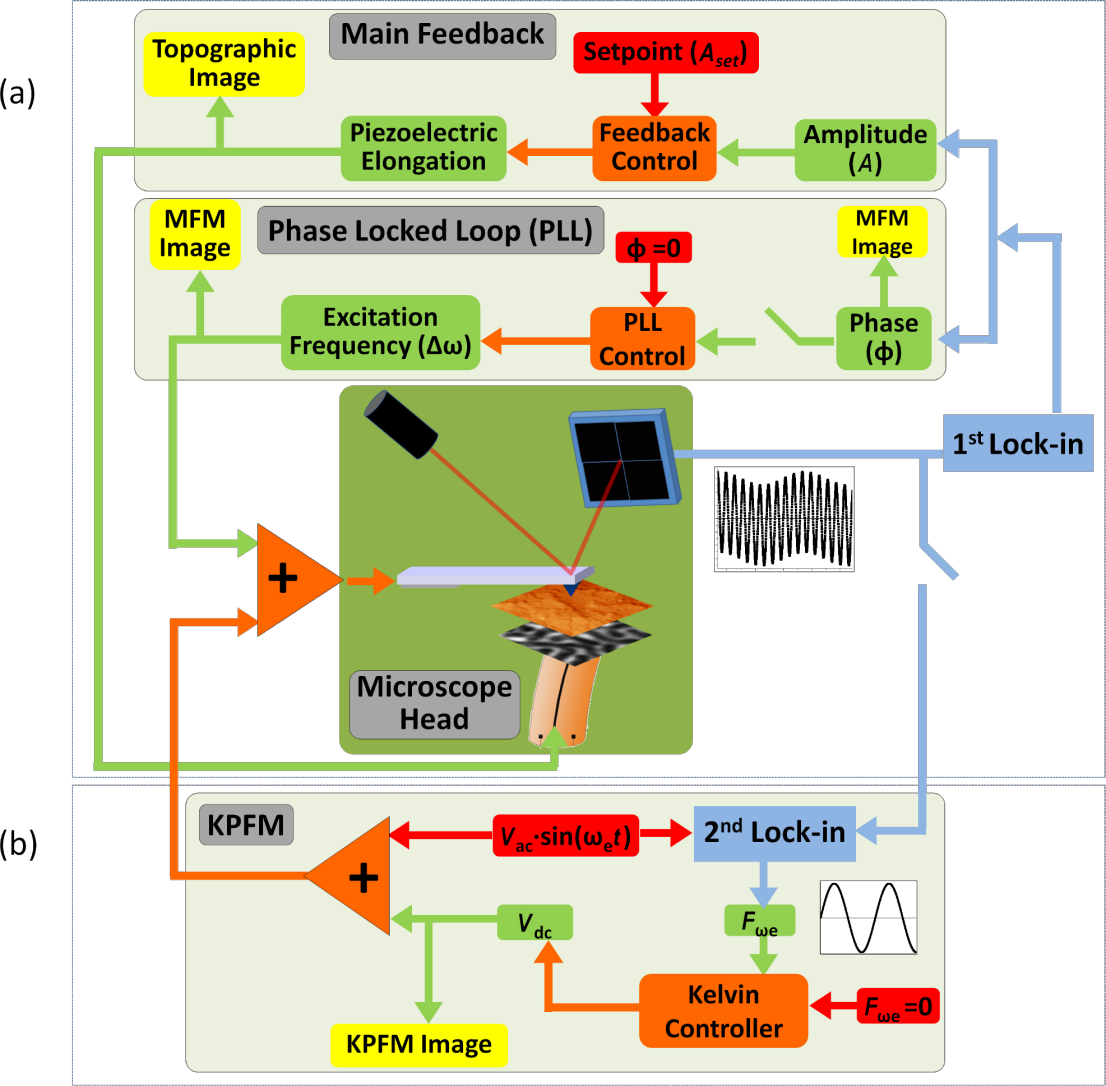
(a) Sketch of the different feedback loops used to perform MFM measurements with PLL system activated and (b) sketch of the MFM/KPFM combined system.

In Figure 4a a schematic of the experimental system is presented. The tip–sample forces can be evaluated simply by measuring the cantilever deflexion. However, dynamic modes are used to improve the sensitivity and resolution of the MFM signal. In any dynamic mode the interaction is evaluated through the force gradient, although the force can be recovered from the curve of frequency shift versus distance [45]. The interpretation of the interaction is more complicated in the case of dynamical modes. The tip–cantilever system oscillates at a certain frequency with a given amplitude. Due to the presence of an interaction between the tip and the sample, the amplitude and the phase of the oscillation change. In our experiments we use a PLL (Phase Locked Loop) system to keep the phase constant while the excitation frequency varies (see the sketch in Figure 4a). Both the amplitude and the frequency shift depend on the force gradient. It is well established that the changes in amplitude are related to dissipative process while changes in the frequency shift are associated with conservative interactions. In the amplitude modulation mode, the amplitude is the main feedback parameter and thus the movement of the piezoelectric is used to build the topography image. The frequency shift changes are recorded at a certain distance to build the magnetic image, thus the MFM images were obtained in the so-called “retrace mode”. During the first scan the oscillation amplitude is kept constant as well as the phase of the oscillation (thanks to the PLL feedback system). The retrace scan is then performed at a selected tip–sample distance, following the topography recorded in the first scan (i.e., with the main feedback switched off).

The frequency shift results from a convolution between the tip–sample force gradient and a weight function. For low oscillation amplitudes, the frequency shift of the cantilever, at a retrace distance large enough to avoid van der Waals interactions, is proportional to the total force gradient (that can be composed of magnetic and/or electrostatic interactions).

The experiments in the present work were performed in ambient conditions, in the non-contact dynamic mode (with low amplitude modulation) and with the PLL feedback activated. In addition, KPFM [17] was used in combination with MFM to adjust the tip bias voltage to minimize electrostatic forces between the tip and the sample at every point on the sample (Figure 4b). In both of the scans (main scan and retrace mode), the normal force, amplitude, phase, frequency shift and surface potential (in the KPFM mode) signals can be recorded simultaneously.

In KPFM, an ac bias voltage (*V*_ac_ sin(ω_e_*t*), where *V*_ac_ = 0.5 V and ω_e_ = 7 kHz) is added to the *V*_dc_ bias voltage. In order to cancel the electrostatic interaction between the tip and the sample, the component of the force that oscillates with *F*_e_ (ω_e_) is nullified by applying the appropriate *V*_dc_ at each tip position; this is the output of the Kelvin feedback.

## Supporting Information

File 1 Topography of the nanostructure.

File 2 MFM images.
