# Supplementary material for: Distinguishing magnetic and electrostatic interactions by a Kelvin probe force microscopy–magnetic force microscopy combination
Source: Beilstein J Nanotechnol. 2011 Sep 7;2:552–60. doi: 10.3762/bjnano.2.59 (PMC3190625; doi:10.3762/bjnano.2.59)

Supporting Information

for

Distinguishing magnetic and electrostatic interactions by a Kelvin probe force microscopy–magnetic force microscopy combination

Miriam Jaafar[1,2], Oscar Iglesias-Freire[1], Luis Serrano-Ramón[3,4], Manuel Ricardo Ibarra[3,4,5], Jose Maria de Teresa[3,4,5] and Agustina Asenjo[*1]

Address: [1]Instituto de Ciencia de Materiales de Madrid, CSIC, Madrid, 28049, Spain; [2]Dpto. Física de la Materia Condensada, Universidad Autónoma de Madrid, Madrid, 28049, Spain; [3]Instituto de Ciencia de Materiales de Aragón, Universidad de Zaragoza-CSIC, Zaragoza, 50009, Spain; [4]Dpto. Física de la Materia Condensada, Universidad de Zaragoza-CSIC, Zaragoza, 50009, Spain and [5]Laboratorio de Microscopías Avanzadas (LMA), Instituto de Nanociencia de Aragón (INA), Universidad de Zaragoza, Zaragoza, 50018, Spain

Email: Agustina Asenjo - aasenjo@icmm.csic.es

* Corresponding author

Topography of the nanostructure

The electrostatic interaction can have a remarkable effect on the topography images in certain kind of samples (see [42]). In our case, due to the height of the structures this effect is not significant. To verify this assumption we present here the topography of the same nanostructure obtained with (a) and without (b) KPFM. As shown in Figure (c), the height difference between both profiles is lower than 1 nm.

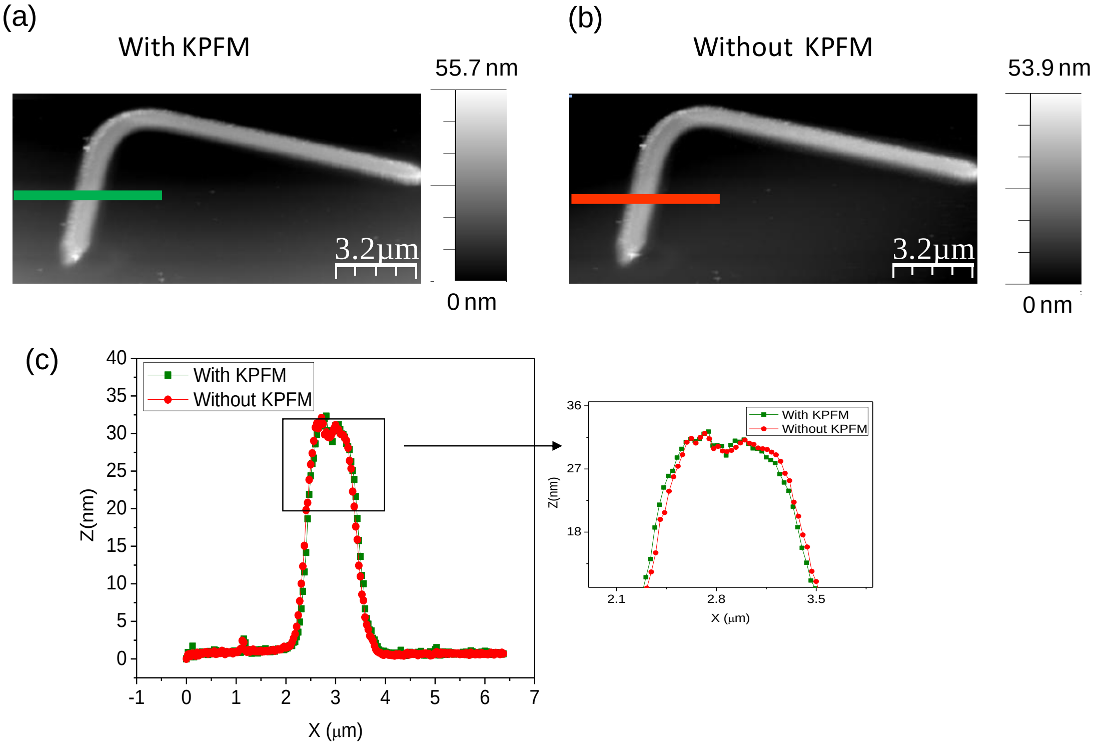

Supplement: File 1 — Topography of the nanostructure. [file Beilstein_J_Nanotechnol-02-552-s001.pdf]
