# Supplementary material for: Distinguishing magnetic and electrostatic interactions by a Kelvin probe force microscopy–magnetic force microscopy combination
Source: Beilstein J Nanotechnol. 2011 Sep 7;2:552–60. doi: 10.3762/bjnano.2.59 (PMC3190625; doi:10.3762/bjnano.2.59)

Supporting Information

for

Distinguishing magnetic and electrostatic interactions by a Kelvin probe force microscopy–magnetic force microscopy combination

Miriam Jaafar[1,2], Oscar Iglesias-Freire[1], Luis Serrano-Ramón[3,4], Manuel Ricardo Ibarra[3,4,5], Jose Maria de Teresa[3,4,5] and Agustina Asenjo[*1]

Address: [1]Instituto de Ciencia de Materiales de Madrid, CSIC, Madrid, 28049, Spain; [2]Dpto. Física de la Materia Condensada, Universidad Autónoma de Madrid, Madrid, 28049, Spain; [3]Instituto de Ciencia de Materiales de Aragón, Universidad de Zaragoza-CSIC, Zaragoza, 50009, Spain; [4]Dpto. Física de la Materia Condensada, Universidad de Zaragoza-CSIC, Zaragoza, 50009, Spain and [5]Laboratorio de Microscopías Avanzadas (LMA), Instituto de Nanociencia de Aragón (INA), Universidad de Zaragoza, Zaragoza, 50018, Spain

Email: Agustina Asenjo - aasenjo@icmm.csic.es

* Corresponding author

MFM images

Variable Field MFM images of a single "L-shaped" nanostructure. During the magnetic imaging process the electrostatic force is compensated by Kelvin Probe Force Microscopy (inset images).The magnetic field is continuously applied in plane direction (see arrows): (a) as-prepared under 0 Oe; (b) −6 Oe; (c) -60 Oe; (d) +100 Oe; (e) +180 Oe and (f) 0 Oe. Image parameters: A = 8.5 nm, ω_0 = 73.83 Hz and a scan rate of 0.74 Hz. The color scale of the images is 5 Hz for the MFM and 0.7 V for the surface potential. Notice that we can follow the magnetization process of this structure from a complex domain configuration (a) to a single domain state (c and f).

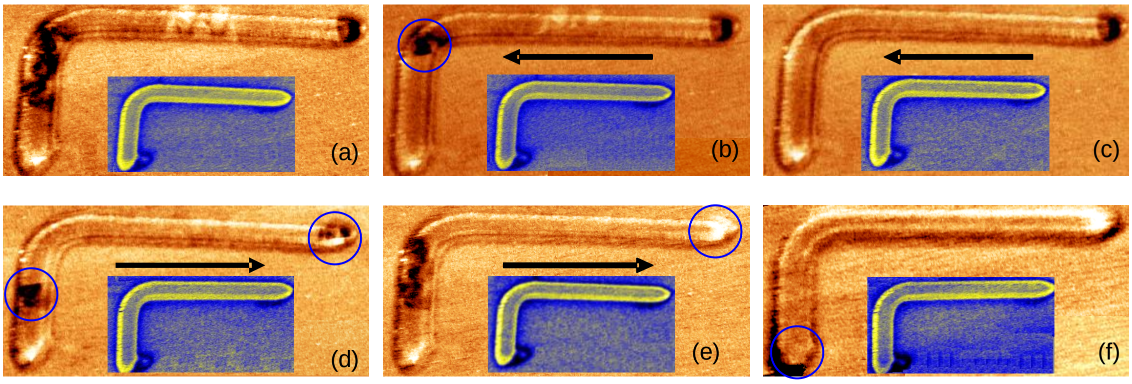

Supplement: File 2 — MFM images. [file Beilstein_J_Nanotechnol-02-552-s002.pdf]
